# Impact of Bromocriptine-QR Therapy on Glycemic Control and Daily Insulin Requirement in Type 2 Diabetes Mellitus Subjects Whose Dysglycemia Is Poorly Controlled on High-Dose Insulin: A Pilot Study

**DOI:** 10.1155/2015/834903

**Published:** 2015-04-28

**Authors:** Erin D. Roe, Bindu Chamarthi, Philip Raskin

**Affiliations:** ^1^University of Texas Southwestern Medical Center, Dallas, TX 75235, USA; ^2^Brigham and Women's Hospital and Harvard Medical School, Boston, MA 02115, USA

## Abstract

*Background*. The concurrent use of a postprandial insulin sensitizing agent, such as bromocriptine-QR, a quick release formulation of bromocriptine, a dopamine D2 receptor agonist, may offer a strategy to improve glycemic control and limit/reduce insulin requirement in type 2 diabetes (T2DM) patients on high-dose insulin. This open label pilot study evaluated this potential utility of bromocriptine-QR. *Methods*. Ten T2DM subjects on metformin (1-2 gm/day) and high-dose (TDID ≥ 65 U/day) basal-bolus insulin were enrolled to receive once daily (morning) bromocriptine-QR (1.6–4.8 mg/day) for 24 weeks. Subjects with at least one postbaseline HbA_1c_ measurement (*N* = 8) were analyzed for change from baseline HbA_1c_, TDID, and postprandial glucose area under the curve of a four-hour mixed meal tolerance test (MMTT). *Results*. Compared to the baseline, average HbA_1c_ decreased 1.76% (9.74 ± 0.56 to 7.98 ± 0.36, *P* = 0.01), average TDID decreased 27% (199 ± 33 to 147 ± 31, *P* = 0.009), and MMTT AUC_60–240_ decreased 32% (*P* = 0.04) over the treatment period. The decline in HbA_1c_ and TDID was observed at 8 weeks and sustained over the remaining 16-week study duration. *Conclusion*. In this study, bromocriptine-QR therapy improved glycemic control and meal tolerance while reducing insulin requirement in T2DM subjects poorly controlled on high-dose insulin therapy.

## 1. Introduction

Maintenance of good glycemic control in type 2 diabetes mellitus (T2DM) patients typically becomes progressively more difficult as the duration of disease lengthens as a result of continuing decline in the capacity of the pancreatic beta cells for appropriate glucose stimulated insulin release, in the presence of insulin resistance [1, 2]. While hyperglycemia in patients with T2DM may be initially managed with oral antidiabetes medications alone, added insulin therapy often becomes necessary with longer duration of disease. This progressive decline in beta cell function amidst a background of insulin resistance, which can be severe in many T2DM patients, particularly with the common concomitant presence of obesity, ultimately results in the need for high doses of insulin in many such patients. While insulin is an effective treatment for hyperglycemia, it carries the potential for undesirable side effects such as hypoglycemia and weight gain, which in turn can lead to a worsening ability to manage diabetes [3–8]. Moreover, chronic high-dose insulin therapy can be difficult to manage from a practical perspective, with inherent challenges related to administration of large doses of insulin, multiple injections, and large volume injections (which could interfere with absorption of the insulin, thus leading to submaximal effect of the administered insulin dose and also adversely impacting patient comfort and compliance) [8–11]. A plausible approach to treating such patients with poor beta cell function and on high-dose daily insulin may be the use of an agent with unique insulin sensitizing properties to improve glycemic control and concurrently reduce the daily insulin requirement. However, the available options for such insulin sensitizing agents that are safe and effective are very limited. The insulin sensitizer, pioglitazone, has been demonstrated to improve glycemia in insulin-requiring T2DM patients [12–15]; however use of this agent in this setting is associated with significant increased risk for congestive heart failure in addition to weight gain and edema [15–20]. Alternatively, circadian-timed, morning administration of bromocriptine-QR, a quick-release, high absorbing formulation of a potent dopamine D2 receptor agonist, that is approved in the US for the treatment of T2DM appears to be a unique insulin sensitizing therapy with a good safety profile, without risk of weight gain or hypoglycemia and with a potential to reduce adverse cardiovascular risk [21–23]. Available evidence suggests that bromocriptine-QR is a postprandial-weighted insulin sensitizer, promoting glucose disposal following a meal, oral glucose tolerance test, or hyperglycemic-euglycemic clamp [24–27]. Several neurophysiological studies have implicated a role for a diminished circadian peak of dopaminergic activity at the biological clock (suprachiasmatic nucleus [SCN] of the hypothalamus) in the development of insulin resistance [28–34]. Timed bromocriptine-QR administration to restore this diminished circadian peak of dopaminergic activity improves insulin sensitivity [24–27]. Preclinical studies suggest that such effects are mediated via a circadian-time-dependent effect to ameliorate aberrations in hypothalamic activities that potentiate insulin resistance and loss of appropriate fuel sensing mechanisms [28–34]. In humans, this daily peak central dopaminergic activity is believed to occur in the morning circa awakening and to be diminished in T2DM [24, 35]. Consequently, we hypothesized that an additive or supra-additive interaction may exist between bromocriptine-QR and basal plus prandial insulin therapy, with bromocriptine-QR providing the enhanced responsiveness to the exogenous prandial insulin therapy to produce greater postprandial blood glucose lowering with lower insulin requirement. We therefore explored in a small open label pilot study the potential utility of morning bromocriptine-QR therapy to improve glycemic control and reduce daily insulin requirement in T2DM subjects on high-dose (≥65 U/day) insulin therapy that incorporates a prandial insulin administration.

## 2. Methods

### 2.1. Subjects and Study Design

Ten subjects with T2DM treated with high-dose insulin [total daily insulin dose (TDID) ≥ 65 units/day] and metformin (1-2 g in divided doses) were recruited from the outpatient primary care and diabetes clinics at Parkland Memorial Hospital or through self-referral at the Clinical Diabetes Office at the University of Texas Southwestern Medical Center in Dallas, TX, and assigned to bromocriptine-QR therapy (see below for dosing regimen). Patients between ages 30 and 65 years with the clinical diagnosis of T2DM on multiple daily injections of insulin (MDI insulin) plus metformin and HbA_1c_ between 7.5 and 12.0% were eligible. Major exclusion criteria for study participation were pregnancy, lactation, type 1 diabetes, elevated serum creatinine over 1.5 mg/dL, elevated liver function tests above 3-fold the upper limit reference range, and a recent history of substance abuse. In addition, patients with a risk of hypotension, recent blood donation in the past 30 days, syncopal migraines, gastroparesis, autonomic neuropathy, hypoglycemia unawareness, uncontrolled mental illness and/or psychosis, and variable sleep patterns (e.g., shift workers) were excluded. Five additional patients with similar demographics as the bromocriptine-QR treated group were recruited under the same study protocol only as a reference for glycemic control achievable only by increasing insulin dose (i.e., absent bromocriptine-QR therapy). In this pilot study design, this reference group was not randomized nor placebo-treated nor able to serve as an active comparator for efficacy due to allowance of insulin dose change during the study and of too small N to serve as a control group for noninferiority testing. The study protocol was approved by the institutional review board (IRB) of the University of Texas Southwestern Medical Center, Dallas, TX, and all participants provided written informed consent prior to enrollment. The study protocol was registered with ClinicalTrials.gov (Identifier: NCT01474018).

### 2.2. Treatment Paradigm

Patients continued baseline treatment with metformin (1-2 gm in divided doses) and MDI insulin (either premixed human 70/30 insulin twice daily or basal-bolus regimen with basal insulin glargine [rDNA origin; Lantus, Sanofi, Paris, France] and insulin aspart [rDNA origin; Novolog, Novo Nordisk, Bagsvaerd, Denmark] at each meal). Bromocriptine-QR was titrated weekly according to a schedule used in previous studies and detailed on the FDA-approved package insert; 1 tablet (0.8 mg) is taken within 2 hours of waking for the first week and each week thereafter; an additional tablet is added until a maximum tolerated dose of two to six tablets (1.6 to 4.8 mg, resp.) during week six was achieved. Patients were contacted by phone each week during the titration period to inquire about hypoglycemia and potential side effects, primarily nausea, headache, and dizziness. If intolerable side effects were encountered, the patient was instructed to reduce the dose to the highest previously tolerated dose and continue taking this dose through the remainder of the 24-week trial. Metformin dose was held constant. Insulin was titrated according to good clinical practice to target an HbA_1c_ ≤ 7.0%, while minimizing hypoglycemia.

### 2.3. Measurements

HbA_1c_ levels and total daily insulin dose requirements were assessed at enrollment and weeks 8, 16, and 24. HbA_1c_ was measured using high performance liquid chromatography in the UTSW Clinical Diabetes Laboratory. At baseline and week 24, a four-hour mixed meal tolerance test (MMTT) was performed in the fasting state with antidiabetic treatments withheld prior to and during the MMTT to evaluate the impact of bromocriptine-QR therapy upon fasting and postprandial blood glucose levels. Antidiabetes medications were withheld prior to and during the MMTT to assess the impact of bromocriptine-QR on endogenous insulin-mediated glucose disposal without interference/confounding of results from a potentially changed exogenous insulin dose requirement during the study period. A four-hour challenge was selected to better quantify the glucose profile which does not normalize within 3 hours in patients with T2DM. A mixed carbohydrate/fat meal was simulated with Boost concentrate 1 gm/kg carbohydrate equivalent ingested over 5 minutes. Glucose levels were measured at 0, 15, 30, 60, 90, 120, 150, 180, and 240 minutes following ingestion. The area under the curve (AUC) for postprandial glucose response [total (0–240 minutes) and postabsorptive (60–240 minutes)] was calculated.

### 2.4. Statistics

Statistical analyses were performed to assess within group pre- versus posttreatment differences. Analyses of the study endpoints used the modified intention-to-treat population of subjects defined as those having at least one postinitiation HbA_1c_ data point utilizing the last observation carry forward method for missing data. Statistical analyses were performed using SPSS Version 19.0 (Armonk, NY: IBM Corp.). The change over time in the HbA_1c_ level and the total daily insulin dose requirement were analyzed by repeated measures analysis of variance (ANOVA). Paired, two-tailed *t*-tests were performed for pre- versus post-8- and 24-week comparisons of HbA_1c_, total daily insulin dose requirement, and the 24-week AUC of postprandial (0–240 and 60–240 minutes) blood glucose during the MMTT. Statistical significance was accepted at the *P* < 0.05 level. Data are presented as mean and standard error of the mean (SEM).

## 3. Results

Of the 10 subjects recruited, two withdrew consent prior to the first HbA_1c_ data point assessment and a third patient was lost to follow-up after the week 8 visit assessment, while on 1.6 mg/day of bromocriptine-QR. Seven subjects completed the study. Of the seven subjects that completed the study, five achieved the maximum bromocriptine-QR dose of 4.8 mg daily and 2 subjects stopped dose titration at 1.6 mg daily due to nausea and headaches and continued this dose for the duration of the study. Statistical analyses were performed on the 8 subjects with a postinitiation HbA_1c_ value. All 5 patients in the reference group completed the study. The study subjects were predominantly black and Hispanic and obese. The baseline demographics of the study subjects are displayed in Table 1.

**Figure 1:**
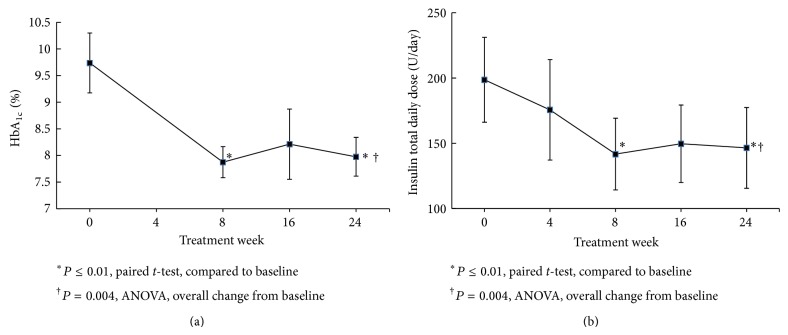
Effect of bromocriptine-QR therapy on HbA_1c_ (Panel (a)) and total daily insulin dose (TDID) (Panel (b)) over 24 weeks in subjects on metformin plus high-dose basal-bolus insulin therapy at baseline.

**Figure 2:**
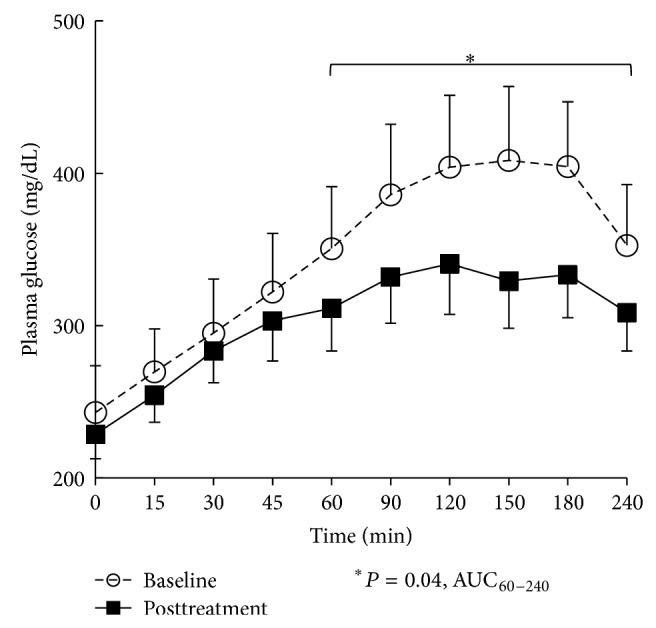
Effect of bromocriptine-QR therapy on mixed meal tolerance test postprandial glucose area under the curve (AUC) over 24 weeks in subjects on metformin plus high-dose basal-bolus insulin therapy at baseline.

There was an overall significant decrease in HbA_1c_ (*P* = 0.004, ANOVA) (Figure 1(a)) and total daily insulin dose (*P* = 0.001, ANOVA) (Figure 1(b)) over the 24-week bromocriptine-QR treatment period. Following 24 weeks of bromocriptine-QR therapy, the average HbA_1c_ decreased by 1.76% compared to the baseline (from 9.74 ± 0.56 to 7.98 ± 0.36, *P* = 0.01) (Figure 1(a)). Two of the 8 patients achieved an HbA_1c_ less than 7.0% (from 8.3% to 6.9% and 11.3 to 6.7%, resp.). Furthermore, this improvement in HbA_1c_ was accompanied by a concurrent reduction in the average daily insulin requirement by 27% (TDID from 199 ± 33 to 147 ± 31, *P* = 0.009) (Figure 1(b)). The decline in HbA_1c_ and in the TDID were observed at 8 weeks of treatment with average A1c decreasing by 1.86% (from 9.74 ± 0.56 to 7.88 ± 0.29, *P* = 0.01) with a concurrent 28.6% reduction in average daily insulin requirement (TDID from 199 ± 33 to 142 ± 27, *P* = 0.01) and were sustained over the remaining 16-week study duration (Figures 1(a) and 1(b)). The bromocriptine-QR induced improvement in HbA_1c_ was associated with a significant reduction in mixed meal tolerance test postprandial glucose AUC tested during the postabsorptive phase (AUC_60–240_, 32% reduction, *P* = 0.04) (Figure 2). The MMTT AUC_0–240_ also showed a trend of reduction in glucose levels that approached but did not achieve statistical significance (29% reduction, *P* = 0.057). There was no significant difference from baseline in the fasting glucose checked at initiation of the MMTT (conducted in the absence of any antidiabetes medications on the day of testing) (fasting glucose 243 ± 31 mg/dL at baseline versus 229 ± 16 mg/dL at 24 weeks).

In contrast, in the reference group, where the treat-to-target goal could be achieved only by raising the insulin dose, a nonstatistically significant 1.08% A1c drop from baseline (from 9.68 ± 0.74 to 8.6 ± 0.62; *P* = 0.19) with no change in fasting or postprandial glucose response during the mixed meal tolerance test was observed over the 24-week study period, while subjects increased the average total daily insulin dose by 26% from 105 ± 41 to 135 ± 49 units.

## 4. Discussion

This pilot study is the first demonstration of an effect of morning bromocriptine-QR therapy to improve glycemic control while enabling a reduction in total daily insulin dose requirement in T2DM subjects whose glycemia was inadequately controlled (HbA_1c_ > 7.5%) on metformin plus high-dose (TDID insulin ≥ 65 units/day) MDI insulin therapy. Addition of bromocriptine-QR therapy to high-dose basal plus prandial insulin regimens resulted in a significant 1.76% HbA_1c_ reduction with a concurrent 27% reduction in daily insulin dose requirement. Such a response to bromocriptine-QR was also coupled to a 32% improvement in the MMTT glucose levels conducted in the absence of any insulin dosing. Contrariwise, increasing the daily insulin dose by 26% from 105 to 132 U/day was without significant improvement in glycemic control in the reference group. Although not specifically tested, these findings support a potential insulin sensitizing effect of bromocriptine-QR in this subject population, which is in agreement with previous preclinical and clinical studies suggesting a postprandial-weighted insulin sensitizing effect of this therapy [24–27, 36, 37]. It should be noted that, in the present study, the MMTT was conducted in the absence of any antidiabetes agent including insulin on the day of the test. Given previous study results demonstrating bromocriptine-QR's effect to improve postprandial insulin sensitivity, its effect on the MMTT would be expected to have been even greater if conducted in the presence of exogenously administered prandial insulin even at its reduced dose after 24 weeks of bromocriptine-QR therapy. In agreement with the present findings and this tenet are the observations that timed bromocriptine therapy improved maximally insulin stimulated glucose disposal during a euglycemic-hyperinsulinemic clamp in severely insulin resistant T2DM subjects on oral antidiabetes agents [25]. Moreover, in insulin resistant animals, the effect of exogenous insulin administration on glucose disposal and glucose tolerance AUC glucose and insulin were each markedly enhanced in animals pretreated with timed daily bromocriptine administration for a two-week period. Such bromocriptine-QR treatment in animals also improves glucose disposal during a hyperglycemic-hyperinsulinemic clamp [26, 27, 36, 37]. By extension, combination of bromocriptine-QR therapy with prandial insulin secretagogues, such as the incretin-mimetics, may also produce similar additive or supra-additive effects as indicated in preclinical studies [38].

Available evidence suggests that timed bromocriptine therapy improves insulin sensitivity and dysglycemia by correcting hypothalamic activity aberrations that potentiate insulin resistance ([30], reviewed in [31]). In normal insulin sensitive states, hypothalamic fuel sensing neurons detect the meal-associated rise in plasma and brain levels of glucose and free fatty acids and, via the neuroendocrine axis, send signals to the peripheral tissues to improve insulin-stimulated glucose disposal, reduce hepatic glucose output, and reduce adipose tissue lipolysis [39–43]. However, in insulin resistant states, such hypothalamic fuel sensing is diminished and consequently these hypothalamic directed responses to produce the above effects in the periphery are also diminished. Resultantly, such hypothalamic aberrations are causative in the insulin resistance and contribute to postprandial hyperglycemic dysglycemia. Importantly, recent studies indicate a role for diminished hypothalamic dopaminergic activity in such aberrant hypothalamic fuel sensing [28, 30, 31, 34, 36, 37, 44–46].

Neurophysiological studies of hypothalamic regulation of fuel metabolism in normal and insulin resistant states indicate that central dopaminergic activities modulate insulin sensitivity as follows. In insulin resistant states, the normal daily peak in dopaminergic activity at the biological clock (hypothalamic SCN) is diminished and reinstating this peak with systemic, central, or direct (to the SCN itself) dopaminergic agonist reverses the insulin resistance ([28, 36, 37, 44, 45], Cincotta and Luo unpublished data). It has been observed that such reductions in hypothalamic dopaminergic activity in insulin resistant states are coupled to elevations of noradrenergic and serotonergic activities at the ventromedial hypothalamus (VMH) and elevated Neuropeptide Y and corticotropin releasing hormone levels at the hypothalamic paraventricular nucleus (PVN), in which neurophysiological alterations have been shown to markedly potentiate insulin resistance in part via a concurrent loss of appropriate hypothalamic fuel sensing (as discussed above) and an elevation of sympathetic tone in the periphery [30–34, 46]. Timed daily administration of bromocriptine to reestablish the daily peak in SCN dopaminergic activity also reverses the VMH and PVN abnormalities described above [30, 37, 46] and improves postprandial insulin resistance (even via its intracerebroventricular administration) [26, 30, 45]. Additionally, a large and growing body of evidence implicates functional roles for decreased mesolimbic dopamine activity in the onset of insulin resistance [47–52] and this neural center is also under the influence of the SCN (reviewed in [53]). Restoration of the circadian peak in central nervous system (hypothalamic) dopaminergic neuronal activity may represent a potential therapeutic target to improve glycemic control in T2DM patients requiring high-dose basal-bolus insulin therapy.

Limitations of this study include the small sample size and no direct measure of insulin sensitivity or beta cell function. However, the significant findings noted in this small pilot study indicate that a trial with a larger sample size and assessing beta cell function and insulin sensitivity is warranted.

In conclusion, bromocriptine therapy in T2DM patients on high-dose insulin and with poor glycemic control improved glycemic control and meal tolerance while simultaneously allowing for a reduction in the daily insulin dose requirement.

## Figures and Tables

**Table 1:** Baseline demographics of the study subjects.

	Bromocriptine-QR treatment group (*N* = 8)	Reference group (*N* = 5)
Age (years)	46 ± 3	54 ± 3
Gender (% female)	88	40
Race	63% B; 25% H; 12% W	60% B; 40% H; 0% W
Weight (lbs)	252.3 ± 20	227.2 ± 17.4
BMI	46.2 ± 5.9	37.1 ± 5.3^*^
Duration of diabetes (years)	13.7 ± 2.2	12.6 ± 3.5
HbA_1c_	9.74 ± 0.56	9.68 ± 0.74
Total daily dose of insulin (units)	199 ± 33	105 ± 41

^*^Mean does not include one double amputee subject.

B: black; H: Hispanic; W: white; BMI: body mass index.

## References

[1] Defronzo R. A. (2009). From the triumvirate to the ominous octet: a new paradigm for the treatment of type 2 diabetes mellitus. *Diabetes*.

[2] Ferrannini E., Gastaldelli A., Miyazaki Y., Matsuda M., Mari A., DeFronzo R. A. (2005). *β*-cell function in subjects spanning the range from normal glucose tolerance to overt diabetes: a new analysis. *Journal of Clinical Endocrinology and Metabolism*.

[3] Khunti K., Davies M., Majeed A., Thorsted B. L., Wolden M. L., Paul S. K. (2015). Hypoglycemia and risk of cardiovascular disease and all-cause mortality in insulin-treated people with type 1 and type 2 diabetes: a cohort study. *Diabetes Care*.

[4] Heller S. R., Choudhary P., Davies C. (2007). Risk of hypoglycaemia in types 1 and 2 diabetes: effects of treatment modalities and their duration. *Diabetologia*.

[5] McNay E. C., Teske J. A., Kotz C. M. (2013). Long-term, intermittent, insulin-induced hypoglycemia produces marked obesity without hyperphagia or insulin resistance: a model for weight gain with intensive insulin therapy. *American Journal of Physiology—Endocrinology and Metabolism*.

[6] Pontiroli A. E., Miele L., Morabito A. (2011). Increase of body weight during the first year of intensive insulin treatment in type 2 diabetes: Systematic review and meta-analysis. *Diabetes, Obesity and Metabolism*.

[7] Russell-Jones D., Khan R. (2007). Insulin-associated weight gain in diabetes—causes, effects and coping strategies. *Diabetes, Obesity and Metabolism*.

[8] Ross S. A., Tildesley H. D., Ashkenas J. (2011). Barriers to effective insulin treatment: the persistence of poor glycemic control in type 2 diabetes. *Current Medical Research and Opinion*.

[9] Hildebrandt P. (1991). Subcutaneous absorption of insulin in insulin-dependent diabetic patients. Influence of species, physico-chemical properties of insulin and physiological factors. *Danish Medical Bulletin*.

[10] Heinemann L. (2002). Variability of insulin absorption and insulin action. *Diabetes Technology and Therapeutics*.

[11] Binder C., Lauritzen T., Faber O., Pramming S. (1984). Insulin pharmacokinetics. *Diabetes Care*.

[12] Mattoo V., Eckland D., Widel M. (2005). Metabolic effects of pioglitazone in combination with insulin in patients with type 2 diabetes mellitus whose disease is not adequately controlled with insulin therapy: results of a six-month, randomized, double-blind, prospective, multicenter, parallel-group study. *Clinical Therapeutics*.

[13] Davidson J. A., Perez A., Zhang J., Pioglitazone 343 Study Group (2006). Addition of pioglitazone to stable insulin therapy in patients with poorly controlled type 2 diabetes: results of a double-blind, multicentre, randomized study. *Diabetes, Obesity and Metabolism*.

[14] Charbonnel B., DeFronzo R., Davidson J. (2010). Pioglitazone use in combination with insulin in the prospective pioglitazone clinical trial in macrovascular events study (PROactive19). *Journal of Clinical Endocrinology and Metabolism*.

[15] Clar C., Royle P., Waugh N. (2009). Adding pioglitazone to insulin containing regimens in type 2 diabetes: systematic review and meta-analysis. *PLoS ONE*.

[16] Hartung D. M., Touchette D. R., Bultemeier N. C., Haxby D. G. (2005). Risk of hospitalization for heart failure associated with thiazolidinedione therapy: a medicaid claims-based case-control study. *Pharmacotherapy*.

[17] Chaggar P. S., Shaw S. M., Williams S. G. (2009). Thiazolidinediones and heart failure. *Diabetes and Vascular Disease Research*.

[18] Giles T. D., Miller A. B., Elkayam U., Bhattacharya M., Perez A. (2008). Pioglitazone and heart failure: results from a controlled study in patients with type 2 diabetes mellitus and systolic dysfunction. *Journal of Cardiac Failure*.

[19] Scheen A. J. (2004). Combined thiazolidinedione-insulin therapy: should we be concerned about safety?. *Drug Safety*.

[20] http://www.accessdata.fda.gov/drugsatfda_docs/label/2011/021073s043s044lbl.pdf.

[21] Gaziano J. M., Cincotta A. H., O'Connor C. M. (2010). Randomized clinical trial of quick-release bromocriptine among patients with type 2 diabetes on overall safety and cardiovascular outcomes. *Diabetes Care*.

[22] Gaziano J. M., Cincotta A. H., Vinik A., Blonde L., Bohannon N., Scranton R. (2012). Effect of bromocriptine-QR (a quick-release formulation of bromocriptine mesylate) on major adverse cardiovascular events in type 2 diabetes subjects. *Journal of the American Heart Association*.

[23] Vinik A. I., Cincotta A. H., Scranton R. E., Bohannon N., Ezrokhi M., Gaziano J. M. (2012). Effect of Bromocriptine-QR on glycemic control in subjects with uncontrolled hyperglycemia on one or two oral anti-diabetes agents. *Endocrine Practice*.

[24] Scranton R., Cincotta A. (2010). Bromocriptine—unique formulation of a dopamine agonist for the treatment of type 2 diabetes. *Expert Opinion on Pharmacotherapy*.

[25] Pijl H., Ohashi S., Matsuda M. (2000). Bromocriptine: a novel approach to the treatment of type 2 diabetes. *Diabetes Care*.

[26] Ezrokhi M., Luo S., Trubitsyna Y., H. Cincotta A. (2012). Weighted effects of bromocriptine treatment on glucose homeostasis
during hyperglycemic versus euglycemic clamp conditions in insulin
resistant hamsters: bromocriptine as a unique postprandial insulin
sensitizer. *Journal of Diabetes & Metabolism*.

[27] Moore M. C., Smith M., Farmer B., Cherrington A. D. (2014). Timed daily bromocriptine mesylate (BC) administration improves glucose disposal in a canine diet-induced model of impaired glucose tolerance. *Diabetologia*.

[28] Luo S., Luo J., Cincotta A. H. (1999). Suprachiasmatic nuclei monoamine metabolism of glucose tolerant versus intolerant hamsters. *NeuroReport*.

[29] Luo S., Luo J., Meier A. H., Cincotta A. H. (1997). Dopaminergic neurotoxin administration to the area of the suprachiasmatic nuclei induces insulin resistance. *NeuroReport*.

[30] Ezrokhi M., Luo S., Trubitsyna Y., Cincotta A. H. (2014). Neuroendocrine and metabolic components of dopamine agonist amelioration of metabolic syndrome in SHR rats. *Diabetology & Metabolic Syndrome*.

[31] Cincotta A. H., Hansen B., Shafrir E. (2002). Hypothalamic role in insulin resistance and insulin resistance syndrome. *Frontiers in Animal Diabetes Research*.

[32] Luo S., Luo J., Cincotta A. H. (1999). Chronic ventromedial hypothalamic infusion of norepinephrine and serotonin promotes insulin resistance and glucose intolerance. *Neuroendocrinology*.

[33] Cincotta A. H., Luo S., Zhang Y. (2000). Chronic infusion of norepinephrine into the VMH of normal rats induces the obese glucose-intolerant state. *The American Journal of Physiology—Regulatory Integrative and Comparative Physiology*.

[34] Luo S., Ezrokhi M., Trubitsyna Y., Cincotta A. H. (2008). Intrahypothalamic circuitry regulating hypothalamic fuel sensing to induce insulin sensitivity or insulin resistance. *Diabetologia*.

[35] Monti J. M., Monti D. (2007). The involvement of dopamine in the modulation of sleep and waking. *Sleep Medicine Reviews*.

[36] Cincotta A. H., MacEachern T. A., Meier A. H. (1993). Bromocriptine redirects metabolism and prevents seasonal onset of obese hyperinsulinemic state in Syrian hamsters. *The American Journal of Physiology—Endocrinology and Metabolism*.

[37] Luo S., Meier A. H., Cincotta A. H. (1998). Bromocriptine reduces obesity, glucose intolerance and extracellular monoamine metabolite levels in the ventromedial hypothalamus of Syrian hamsters. *Neuroendocrinology*.

[38] Ezrokhi M., Luo S., Trubitsyna T., Cincotta A. H. (2011). Synergism of dopamine agonist plus GLP-1 analog therapy on improvement of glucose intolerance in Syrian hamsters. *Diabetes*.

[39] Pocai A., Lam T. K. T., Obici S. (2006). Restoration of hypothalamic lipid sensing normalizes energy and glucose homeostasis in overfed rats. *Journal of Clinical Investigation*.

[40] Lam T. K. T. (2010). Neuronal regulation of homeostasis by nutrient sensing. *Nature Medicine*.

[41] Breen D. M., Yang C. S., Lam T. K. T. (2011). Gut-brain signalling: how lipids can trigger the gut. *Diabetes/Metabolism Research and Reviews*.

[42] Jordan S. D., Könner A. C., Brüning J. C. (2010). Sensing the fuels: glucose and lipid signaling in the CNS controlling energy homeostasis. *Cellular and Molecular Life Sciences*.

[43] Morgan K., Obici S., Rossetti L. (2004). Hypothalamic responses to long-chain fatty acids are nutritionally regulated. *The Journal of Biological Chemistry*.

[44] Luo S., Zhang Y., Ezrokhi M., Trubitsyna Y., Cincotta A. H. (2014). High-fat feeding abolishes the insulin-sensitizing peak in circadian dopamine activity at the biological clock. *Diabetes*.

[45] Luo S., Liang Y., Cincotta A. H. (1999). Intracerebroventricular administration of bromocriptine ameliorates the insulin-resistant/glucose-intolerant state in hamsters. *Neuroendocrinology*.

[46] Bina K. G., Cincotta A. H. (2000). Dopaminergic agonists normalize elevated hypothalamic neuropeptide Y and corticotropin-releasing hormone, body weight gain, and hyperglycemia in ob/ob mice. *Neuroendocrinology*.

[47] Geiger B. M., Haburcak M., Avena N. M., Moyer M. C., Hoebel B. G., Pothos E. N. (2009). Deficits of mesolimbic dopamine neurotransmission in rat dietary obesity. *Neuroscience*.

[48] Roseberry A. G., Painter T., Mark G. P., Williams J. T. (2007). Decreased vesicular somatodendritic dopamine stores in leptin-deficient mice. *Journal of Neuroscience*.

[49] Davis J. F., Tracy A. L., Schurdak J. D. (2008). Exposure to elevated levels of dietary fat attenuates psychostimulant reward and mesolimbic dopamine turnover in the rat. *Behavioral Neuroscience*.

[50] Geiger B. M., Behr G. G., Frank L. E. (2008). Evidence for defective mesolimbic dopamine exocytosis in obesity-prone rats. *FASEB Journal*.

[51] Rada P., Bocarsly M. E., Barson J. R., Hoebel B. G., Leibowitz S. F. (2010). Reduced accumbens dopamine in Sprague-Dawley rats prone to overeating a fat-rich diet. *Physiology and Behavior*.

[52] Volkow N. D., Wang G.-J., Telang F. (2008). Low dopamine striatal D2 receptors are associated with prefrontal metabolism in obese subjects: possible contributing factors. *NeuroImage*.

[53] Mendoza J., Challet E. (2014). Circadian insights into dopamine mechanisms. *Neuroscience*.

